# The feasibility of identifying health inequalities in social prescribing referrals and declines using primary care patient records

**DOI:** 10.3310/nihropenres.13325.1

**Published:** 2023-01-16

**Authors:** Koser Khan, Rachel Al-Izzi, Alexander Montasem, Clare Gordon, Heather Brown, Joanna Goldthorpe

**Affiliations:** 1 Senior Research Associate, NIHR Applied Research Collaboration NWC, Lancaster University, Lancaster, UK; 2 PhD student, University of Central Lancashire, Preston, PR1 2HE, UK; 3 Senior Lecturer in Social and Behavioural Sciences, School of Medicine, University of Central Lancashire, Prseton, PR1 2HE, UK; 4 Senior Research Fellow, Faculty of Health of Care, University of Central Lancashire, Preston, PR1 2HE, UK; 5 Professor of Health Inequalities, Division of Health Research, Lancaster University, Lancaster, UK; 6 Research Fellow NIHR Applied Research Collaboration NWC, Lancaster University, Lancaster, UK

**Keywords:** social prescribing, health inequalities, representation, GP referrals, decline

## Abstract

**Background:**

Social prescribing (SP) is part of universal personalised care and available to everyone in the UK National Health Service. However, emerging evidence suggests access disparities in social prescribing. This study aimed to investigate the feasibility of accessing and analysing data on social prescribing from primary care records. Our secondary aim was to examine exposure to social prescribing and compare characteristics of patients who decline/accept social prescribing referrals to explore possible health inequalities in access to social prescribing.

**Methods:**

Patient records (n=3086) were extracted from 11 GP practices across Northwest England for accepted, offered and declined social prescribing referrals. Patient demographics collected included sex, age, ethnicity, mental and physical health diagnoses. Patient characteristics in social prescribing referrals were compared to the overall practice population (practice information from Public Health England). Decline and acceptance rates were compared by group (e.g. male/female decline rates).

**Results:**

GP referral data showed inconsistent recording of wider determinants of health and variations in referral patterns on a practice-to-practice basis. Some variables had very poor rates of recording and did not yield useful information. Patient age, sex and mental and physical health conditions were consistently recorded. Other variables such as disability and housing status showed sporadic GP recording across our dataset. Our preliminary findings identified underrepresentation of younger age groups and Asians, and higher declined referrals among individuals with physical health diagnoses.

**Conclusions:**

The differing referral patterns between practices and recording discrepancies meant that many patient factors could not be used to assess trends in social prescribing referrals and declines. Preliminary results suggest that some patient groups may be underrepresented in referral data, however this needs further research and investigation. Consistency is required in social prescribing data recording in primary care. Data on wider determinants is needed to assess variations in referrals and declines and explore inequalities.

## Introduction

Social prescribing (SP) is gaining traction in the UK as a wellbeing intervention and a means to bridge the gap between traditional healthcare and community services. There are many definitions of social prescribing. For the purpose of this paper, we use the Kings Fund definition.

*“Social prescribing, also sometimes known as community referral, is a means of enabling health professionals to refer people to a range of local, non-clinical services. The referrals generally, but not exclusively, come from professionals working in primary care settings, for example, GPs or practice nurses.”*

Different SP models have evolved across England and Wales reflecting local needs, service provider expertise and commissioning contracts. Although SP is accessed almost universally through a GP referral, there is no one standard model ^1^. It is therefore a complex non-medical intervention with numerous interacting components from systems and services to providers and patients ^2, 3^.

General practitioners, nurses, and allied health care professionals can refer patients with a broad range of needs (e.g., reducing loneliness) and clinical conditions (e.g., long-term illness) to a local SP scheme. Social prescribing link workers (SPLWs) are not health care professionals and serve as the core contact for those who have been referred into social prescribing by the health care professional or GP. A SPLW sets up a personalised action plan and connects the referred patient to a range of local activities and community groups that offer practical, social and emotional support. These offers are generally provided by local agencies such as voluntary and community sector organisations (VCS).

Whilst there is considerable evidence to support the use of SP ^4, 5^, a common criticism is a lack of robust evidence on its effectiveness ^6– 10^. SP has been suggested as a tool to reduce health inequalities by supporting individuals in areas of deprivation ^11– 14^. However, little is known on how SP affects health inequalities despite the recent push on SP activities across the UK, particularly within primary care. A recent review recommended more research into the impact of SP on inequalities ^15^. Although SP may improve the social and behavioural determinants of long-term conditions and health inequalities, the opposite may be equally true: health inequalities may be exacerbated when access is affected by socio-economic contexts. Evidence suggests that where interventions, such as SP, focus on individual level changes (for instance, behaviour change) health inequalities may increase ^16^. Research has also pointed to disparities in access, with groups such as young people, men and minority communities less represented in SP service user data ^16– 18^. Again, little is known about why these groups are not accessing health services as readily as others.

Typically, GPs first refer patients to SP. Patients can decline the offer at this referral stage. To gain a better understanding of the relationship between access to SP and the impact of health inequalities we need a better understanding of who is and who is not taking up the offer of SP. One way we can do this is by exploring the characteristics of those patients who self-exclude at the early (referral) stage, as well as those who go on to engage with SP-related services to help us identify specific population groups who do not engage as readily as others.

Our study aimed to investigate the feasibility of accessing and analysing data on SP declines and referrals from primary care records held by GP practices in the Northwest Coast and to gather preliminary evidence on possible health inequalities in the take-up of SP. This involved exploring the data on exposure to SP and comparing patient characteristics of declined or accepted SP referrals to identify any variations across different population groups.

## Methods

GP practices in the Northwest of England were identified through existing NIHR infrastructure (Applied Research Collaboration Northwest Coast and Clinical Research Network Northwest Coast). Purposeful sampling was used to select practices in areas of high deprivation and populations with diverse ethnic groups. As this was a small, low-resource feasibility study it was important to identify practices that would have the capacity to participate, therefore a convenience sampling approach was further applied. Practices that were both formally recording social prescribing data and had practice managers that could support the research were included in the final sample. To align with capacity and provide enough insight into feasibility we aimed to recruit around 10 practices.

11 GP practices participated in the study and completed IRAS Organisation Information Documentation for authorising sharing of practice data. Individual patient consent was not required as patient identifiable information was not included in the dataset.

### Ethics

NHS Health Service Research approval for this study was granted in December 2021 (21/HRA/4891). Approval was also granted by University of Central Lancashire and University of Lancaster. Consent to participate was not applicable as the study used secondary anonymised data and did not involve human subjects.

### Data collection

Data were extracted from11 GP practices across Northwest England over three regions: Liverpool (n=3), East Lancashire (n=4), Blackburn with Darwen (n=1) and Blackpool (n=3) during March 2022. A Clinical Research Network (CRN) lead (GP) developed a query to extract the data from EMIS systems (Liverpool extracted their own data and data from East Lancashire was extracted by a commissioning support unit data quality specialist). All cases were extracted since SP recording began within the practice (most cases between 2019–2022). One practice (practice 8) did not complete data collection and was omitted from analysis.

A standardised search and data extraction strategy was developed in conjunction with a GP representative to extract all patient records with a recorded social prescribing code. These codes were:

- T871691000000100 | Social prescribing offered (finding)
- 871711000000103 | Social prescribing declined (situation)
- 871731000000106 | Referral to social prescribing service (procedure)

Patient variables included in the search are shown in Table 1.

**Table 1.** Patient demographics included in each SP patient case.

Main demographics	Mental ealth	General health	Excluded	Excluded
Age	Mental illness	Respiratory	Marital status	Sexual orientation
Sex	Employment	Diabetes	Interpreter need	Non-english speaking
IMD	Housing	Obesity	Education status	Nationality
Ethnic group	Carer status	Heart Disease	Disability	

Demographics which were excluded from analysis due to high rates of missing data are indicated. Comparison data for overall practice populations (dated 2021) were accessed from practice profiles on Fingertips, Public Health England (May 2022).

### Data analysis

Data were compiled in Microsoft® Excel® (Version 2211) and analysed using IBM® SPSS Statistics (Version 28). Data quality was assessed by identifying data missingness across the identified variables. 3086 total patient cases were available for study, however gaps in recording meant that some fields were excluded altogether, and some others did not include the whole cohort of patient cases. Other fields were only filled in the case of a positive result: mental illness, the four general health categories and carer status. In the health categories missing data indicated no diagnosis has been coded by the patient’s GP.

Missing data in housing, employment, carer status and ethnic group was assessed for patterns between variables and on a practice-to-practice basis. For housing, employment and carer status recording differed on a practice-to-practice basis, suggesting that practice recording habits were a factor.

Data analysed included practice population comparisons and acceptance and decline rates.

***Practice population comparison***. Overall practice population data included total numbers of assigned male and female registered patients split into five-year age groups, the practice Index of Multiple Deprivation (IMD) decile and percentage representation of census ethnic minority groups.

Comparison was drawn between the overall practice population and the SP population. All three SP codes (‘referred’, ‘declined’ and ‘offered’ as outlined in the Data Collection section above) were used to present the total numbers of those who had been ‘exposed’ to social prescribing through their GP. Further information on the ethnic group and IMD comparisons are detailed below under their respective headings.

***Ethnicity***. Comparison practice population data included percentage of each census-based ethnic group (Asian, Black, Mixed, Other). Where an ethnic group’s proportion was less than 1% this was included in a general ‘non-white’ group (approx. 0.9% across all practices) and specific ethnic group was unavailable. This meant comparison was more approximate than for other demographics where whole numbers were available. Additionally, practice percentage of individual ethnic minority groups might have been slightly higher than shown due to the recording of a ‘non-white’ percentage in some practices.

***IMD decile***. Index of Multiple Deprivation decile data was presented at practice level.

***Acceptance/decline rates***. Ratios of acceptance and decline were compared by patient demographic. The three SP codes were used for comparison (declined, offered, referred). The declined code was used where individuals had turned down an offer of referral by their GP. The referred code was used for those referred to the service by their GP. The offered code was used when a patient was offered social prescribing and had neither accepted nor declined initially and therefore described as SP being considered by patient.

Three practices included free text with their SP coding which indicated that in some cases the ‘offered’ code did not reflect the actual situation. The text associated with ten ‘offered’ codes (practice 1: 0/31, practice 2: 1/296, practice 3: 9/250) indicated the patient had actually subsequently declined SP. One additional case was coded as ‘offered’ but rejected by the SPLW as the patient was ‘too young for social prescribing’.

Rates were compared for each demographic using SPSS (Chi-square and Cramers V).

## Results

All results are compiled in Table 2.

**Table 2.** Total numbers and percentages for each demographic under all 3 SP codes and by referrals and declines. The ‘Exposure and practice population’ column shows the total number of cases pulled under all three SP codes (total SP dataset) by whole number and percentage split for the demographic. A comparison to the percentage within the practice population is shown where data is available. Where there is missing data in the dataset this is indicated. The ‘Referral and decline’ rates column show comparisons between demographics and percentages within demographic (i.e. percentage of [male declines]/[male total SP dataset]) Relevant significant results are indicated with ▼ (low) or ▲ (high) and marked in bold. Chi-square with Cramer’s V (p<0.05).

		Exposure and practice population			Referral and decline rates			
Demographic	Category	Exposed (all codes)	Exposed %	Practice %	Referred	Percentage	Declined	Percentage
**Sex**	Male	1287	41.7%	49.5%	441	34.3%	332	25.0%
	Female	1799	58.3%	50.5%	695	38.6%	401	22.3%
**Age**	0–14	22	0.7%	16.3%	16	72.7%	4	18.2%
	15–29	381	12.3%	17.7%	141	37.0%	73	19.2%
	30–44	598	19.4%	19.0%	261	43.6%	91	15.2%
	45–59	772	25.0%	20.0%	319	41.3%	143	18.5%
	60–74	710	23.0%	17.6%	235	33.1%	229	32.3%
	75–89	520	16.9%	8.6%	139	26.7%	160	30.8%
	90+	83	2.7%	0.9%	25	30.1%	23	27.7%
**Ethnic group**	White	2444	88.6%	93.5%	910	37.2%	599	24.5%
	Asian	74	2.7%	4.2%	26	35.1%	13	17.6%
	Black	89	3.2%	0.3%	30	33.7%	**9▼**	**10.1%**
	Mixed	62	2.2%	0.8%	20	32.3%	10	16.1%
	Other	89	3.2%	0.3%	24	27.0%	17	19.1%
	All non- whiten-w	314	11.4%	6.5%	100	31.8%	49	15.6%
**IMD decile**	1	1568	50.8%	50.0%	928	59.2%	398	25.4%
	2	311	10.1%		97	31.2%	76	24.4%
	3	236	7.6%		53	22.5%	20	8.5%
	4	254	8.2%		13	5.1%	15	5.9%
	5	244	7.9%	40.0%	20	8.2%	115	47.1%
	6	100	3.2%		8	8.0%	35	35.0%
	7	171	5.5%	10.0%	5	2.9%	30	17.5%
	8	147	4.8%		8	5.4%	34	23.1%
	9	46	1.5%		1	2.2%	0	0.0%
	10	6	0.2%		0	0.0%	0	0.0%
**Mental illness**	Diagnosis	2374	76.9%		890	37.5%	**486▼**	**20.5%**
	No diagnosis	714	23.1%		246	34.5%	**237▲**	**33.2%**
**Employment** **status**	Employed	32	6.5%		14	43.8%	9	28.1%
	Unemployed	324	66.1%		116	35.8%	**40▼**	**12.3%**
	Retired	134	27.3%		36	26.9%	**39▲**	**29.1%**
	**Total**	**490**						
	Missing data	2596						
**Housing status**	Homeless	58	15.9%		22	37.9%	**2▼**	**3.4%**
	Supported living	198	54.2%		70	35.4%	**54▲**	**27.3%**
	Care/ children’s home	31	8.5%		14	45.2%	6	19.4%
	Private home	78	21.4%		19	24.4%	13	16.7%
	**Total**	**365**						
	Missing data	2721						
**Carer status**	Carer	230	7.5%		85	37.0%	**38▼**	**16.5%**
	Receiving care	123	4.0%		49	39.8%	**10▼**	**8.1%**
	No carer status	2733	88.6%		1002	36.7%	675	42.7%
**General health**	No diagnosis	1507	48.8%		559	37.1%	**292▼**	**19.4%**
	1 Diagnosis	1030	33.4%		377	36.6%	252	24.5%
	2 Diagnoses	397	12.9%		153	38.5%	**121▲**	**30.5%**
	3 Diagnoses	132	4.3%		41	31.1%	**50▲**	**37.9%**
	4 Diagnoses	20	0.6%		6	30.0%	8	40.0%

### Data gaps/quality

In all cases information for the patient’s assigned sex and age was available. For the mental and physical health fields missing data meant no diagnosis had been coded in the patient’s record by the GP. This meant that missing data was treated as absence of illness as in the case of diagnosed mental illness (76.9% n=2374) and no diagnosis of mental illness (23.1% n=714). These medical fields were therefore treated as complete with no true missing data.

Ethnic group was missing from patient records in 10.6% (n=328) cases. The pattern of missing data for ethnic group appeared to be random with similar rates across the practices. All analysis of ethnic group was carried out on the 89.3% (n=2758) where ethnic group was known. The average of all percentages across the ten practices was calculated and used to show comparison in Table 2. White ethnic minorities were not recorded in practice data. Our dataset did contain some white ethnic minority groups including Irish Travellers (n=4), however as we had no comparison data from the practice population, we excluded these from analysis.

Total numbers are shown in Table 2 and missing data where applicable. Data on the desired patient variables related to the wider determinants was not consistently recorded in patient records. Seven variables as highlighted in Table 1 were therefore excluded from the analysis. The disability field was only completed for 1.81% (n=56) of patients in the dataset, highlighting that this type of information may not be routinely collected in primary care.

Fields were excluded where total numbers made up less than 10% (n=308) over the whole dataset, or where subsequent grouping resulted in low numbers. Housing and employment status were included in the results table as they met this threshold but recording was poor for these fields. Missing data in employment status and housing status was 84.9% (n=2596) and 88.17% (n=2721) respectively. Available data therefore only represents a small proportion of the total sample data.

Due to the sporadic nature of GP recording for these variables it is also uncertain if they are an up-to-date reflection of patient status. In the carer status category missing data might mean the recording party is unaware of the patient’s status as a carer/receiving care or that the patient is carrying out this role on an unofficial basis. For this reason, missing data cannot be reliably assumed in all cases to mean a patient is not a carer/receiving care.

Data quality varied between fields. Recording was completed in all cases for the patients’ age, sex and IMD; in most cases for ethnic background (85% n=2758); and in the event of a positive result in the health fields. Data quality was much less robust for the carer, employment and housing status fields, due to missing data, recording uncertainty and the non-medical nature of these fields.

### Preliminary analysis

Preliminary analysis suggests over representation in exposure to social prescribing amongst older age groups compared to the general practice population. However, the analysis also suggests that older age groups are more likely to decline SP. There was some preliminary evidence to suggest higher decline rates in White British people compared to ethnic minority groups and a lower exposure for the Asian ethnic group. In our sample there were more women than men. Those with physical health diagnoses were more likely to decline referral than those without, and those with mental health diagnoses were less likely to decline than those without.

Differences in referral rates were explored in Table 3. There was some variation by locality which will have implications for future research.

**Table 3.** An overview of referral patterns for each of the 10 practices included in the study. Total numbers are shown for the overall practice population and the total patients coded under any of the 3 SP codes (referred, declined, offered). This is then presented as a percentage of the overall practice population. Total numbers and percentages are also provided for each region. Percentage breakdown by SP code is presented for each practice.

	Liverpool			East Lancs				Blackpool			
	practices:			practices:				practices: s			
	1	2	3	4	5	6	7	9	10	11	Total
Practice total	9335	6896	7976	25043	6265	4909	15054	12750	12348	8147	108723
SP total	759	492	543	146	75	136	347	225	244	119	3086
SP practice %	8.13%	7.13%	6.81%	0.58%	1.20%	2.77%	2.31%	1.76%	1.98%	1.46%	
Regional total	24207			51271				33245			108723
Regional SP total	1794			704				588			3086
Regional SP %	7.41%			1.37%				1.77%			
% Referred	44%	28%	26%	75%	76%	68%	34%	25%	28%	19%	
% Offered	4%	60%	46%	23%	20%	21%	59%	61%	62%	67%	
% Declined	52%	12%	28%	3%	4%	12%	7%	14%	10%	13%	

### GP referral patterns

Discrepancies in SP activity were apparent on a practice-by-practice basis. Some practices were much more active than others in offering referral. Distribution of the SP codes differed between practices also. This is shown in Table 3.

## Discussion

This study aimed to assess the feasibility of using data organised against primary care SP codes to explore SP referral patterns and take up. Secondly, the study aimed to examine exposure and referral/decline patterns in patients to give insight into possible health inequalities in accessing SP.

The dataset was relatively simple to access once approvals were in place and provided a broad overview from 10 different practices across the region. This provided a useful insight into referral trends by region, area and by practice. The extent of practice-by-practice and regional variation in our dataset suggests that SP activity is very heterogeneous. However, data quality was insufficient to provide an accurate reflection of referral and declines across the selected patient variables. Practice rates varied for declined, offered and referred codes and it is unclear whether declines by patients are always being recorded by GPs. Repetition of this project with larger datasets might provide more robust evidence on social prescribing referral trends for sex, age, ethnic group and mental and physical health diagnoses. For the wider determinants of health (e.g. disability, employment status etc) with poorer rates, homogeneity and reliability of recording by GPs, repetition is unlikely to yield useful information unless data recording is improved. Our preliminary findings highlight that further research is required to elucidate why GP recording for these variables is poor and what strategies could support better data collection.

Data on housing, carer status and disability for example were generally poorly recorded in the sample. However, practices in our study also showed variation in recording of the demographic data between them, suggesting inconsistencies in data recording within primary care. Moscrop *et al.* 2019 highlight that although an individual’s social and economic circumstances can influence their health care access and outcomes, data relating to these circumstances are not regularly assessed or collected. Collecting such data could improve health equity by enabling a better understanding of who is or is not accessing services and how to better target service provision based on need ^19^.

Our findings demonstrate the need for better data recording in SP as did other research studies exploring the outcomes of SP ^6, 7^. Patient characteristics must be consistently recorded to explore who is or is not being offered social prescribing and those who are not taking up the offer. Without such data, referring organisations may unknowingly be contributing to inequity of access by only referring certain population groups. Watt 2013 stated that it is ‘widely recognised that many health improvement initiatives may have widened inequalities in health as a result of differential uptake by different social groups’ ^20^.

Our preliminary findings from the available data also suggest potential inequalities in uptake of SP, with differences in acceptance and decline rates amongst difference patient demographics that require further investigation. In our sample, younger age groups were underrepresented. Representation also varied between ethnic groups with underrepresentation in the Asian ethnic group. In addition, our findings suggested that users with diagnosed health conditions in the four fields assessed (respiratory, diabetes, obesity, heart disease were more likely to decline referral than those without diagnoses. Such potential variations in uptake are of particular significance to health equity, particularly when such groups may face poor health and additional barriers in accessing services ^16– 18^. The underrepresentation of ethnic minorities in SP has also been highlighted by the ‘Social Prescribing Observatory’, National Academy for Social Prescribing (NASP) and other research reports ^17, 18^.

Future research is needed to identify if these results are reflective of social prescribing referrals more generally. Understanding how GPs make decisions about candidates for referral is important to identify both the appropriateness of referrals and any biases and pre-conceptions that may be impacting certain population groups ^18, 20, 21^.

### Limitations

The heterogeneity in referral patterns and variations in recording may not have given us a full picture of declines however the data has highlighted possible differences in referral and decline rates for some population groups.

Using the practice patient population data ( Fingertips, Public Health England) for comparison was useful in assessing exposure to SP but had some limitations: ethnicity information included only practice percentage by major ethnic group (e.g. 3.2% Asian); IMD was only listed for the practice location: half of the practices in our sample were located in an area of high deprivation which may impact the findings. The SP dataset provided a useful overview of referral patterns but lacked context. Although the practice-to-practice and demographic-based variation in referral patterns was clear, the dataset did not provide any insight into the reasons behind this. More research is needed in this area.

## Conclusion

There is potential to use primary care data on SP to assess who is being referred and who is declining the offer of SP. However, there are concerns that this data is currently not robust enough. A consensus in GP recording of social prescribing codes and patient demographics is required to allow full assessment of patient records for studies of this kind in the future. It is particularly important that more robust data on the wider determinants of health is routinely collected if we are to demonstrate impacts on health inequalities and understand which population groups are benefiting from accessing SP and which may not be. This would allow for assessment of inequalities in referral and uptake of SP and how these may be addressed for specific population groups.

Social prescribing is a universal personalised care scheme which should be available to all and equitable access of the service is an integral part of that. However, our preliminary findings suggest variations may exist in decline and referral rates for SP in primary care amongst some patient population groups, particularly those with long term conditions, ethnic minority groups and younger people. Highlighting the need for further research to assess the actual prevalence of this and explore what factors may be contributing to any variations and how they may be addressed. Exploring referral behaviours will also help identify if any biases are at play and if professionals are indirectly excluding certain population groups.

## Data Availability

The HRA agreement for our study does not allow dissemination of the raw data beyond the primary research team. However, metadata comprising total numbers within our demographic fields is available to readers on request. Please contact the corresponding author for details ( ral-izzi@uclan.ac.uk).
