# Supplementary material for: The feasibility of identifying health inequalities in social prescribing referrals and declines using primary care patient records
Source: NIHR Open Res. 2023 Aug 30;3:1. Originally published 2023 Jan 16. [Version 2] doi: 10.3310/nihropenres.13325.2 (PMC10593349; doi:10.3310/nihropenres.13325.2)
Supplement: Peer review history

# Reviewer report: Reviewer response for version 2

**DOI:** 10.3310/nihropenres.14619.r30334

**Published:** 2023-09-11

**Authors:** Andrea E. Williamson

**Affiliations:** 1 Undergraduate Medical School, College of Medicine, Veterinary and Life Sciences, University of Glasgow, Glasgow, Scotland, UK

I am happy with the changes made.

Is the work clearly and accurately presented and does it cite the current literature?

Yes

If applicable, is the statistical analysis and its interpretation appropriate?

Yes

Are all the source data underlying the results available to ensure full reproducibility?

No

Is the study design appropriate and is the work technically sound?

Yes

Are the conclusions drawn adequately supported by the results?

Partly

Are sufficient details of methods and analysis provided to allow replication by others?

Yes

Reviewer Expertise:

Academic GP, Inclusion Health and tackling health inequalities expertise, experience of using large GP data sets in research.

I confirm that I have read this submission and believe that I have an appropriate level of expertise to confirm that it is of an acceptable scientific standard.

# Reviewer report: Reviewer response for version 2

**DOI:** 10.3310/nihropenres.14619.r30333

**Published:** 2023-09-06

**Authors:** Andrew Moscrop

**Affiliations:** 1 Luther Street Medical Centre, Oxford, England, UK

The authors have put thought and effort into amending their paper. I think it is improved. Many thanks.

Is the work clearly and accurately presented and does it cite the current literature?

Partly

If applicable, is the statistical analysis and its interpretation appropriate?

Yes

Are all the source data underlying the results available to ensure full reproducibility?

No

Is the study design appropriate and is the work technically sound?

Yes

Are the conclusions drawn adequately supported by the results?

Yes

Are sufficient details of methods and analysis provided to allow replication by others?

Yes

Reviewer Expertise:

I feel able to comment on the measuring and monitoring of healthcare inequalities, and on relevant data recording.

I confirm that I have read this submission and believe that I have an appropriate level of expertise to confirm that it is of an acceptable scientific standard.

# Reviewer report: Reviewer response for version 1

**DOI:** 10.3310/nihropenres.14451.r29422

**Published:** 2023-06-23

**Authors:** Andrew Moscrop

**Affiliations:** 1 Luther Street Medical Centre, Oxford, England, UK

This paper demonstrates two important points: despite claims that social prescribing will reduce health inequalities, current patterns of data collection are inadequate to a) support those claims, and b) monitor the equitable distribution of offers and uptake of social prescribing.

 A few comments follow, mostly on presentation and clarity.

 On the Abstract

- I found the abstract not as clear as it could be.
- ‘Exposure to social prescribing’ is ambiguous – it could mean being offered, or actually having experience of social prescribing, or something else.
- I am not sure that the phrase ‘health inequalities in access to social prescribing’ (included in the abstract and elsewhere) is appropriate. Inequalities, yes. But health inequalities, no.
- ‘Underrepresentation of younger age groups and Asians’ is also ambiguous – it might mean that they were not offered, or did not take up an offer.
- The results section of the abstract is a bit repetitive. Perhaps it could be made tighter? For example:
  “GP referral data showed inconsistent recording of health determinants. Patient age, sex and mental and physical health conditions were consistently recorded. Other variables only sporadically recorded by GPs included disability, housing status ( *and name others*).
- Variations in referral patterns were found between GP practices. Our preliminary findings identified underrepresentation of younger age groups and Asians, and higher declined referrals among individuals with physical health diagnoses.”
- The conclusion says that some patient factors could not be used to assess trends – I think this is a very significant finding and the patient factors that could not be used (but which might have been very relevant) should be mentioned specifically – disability, carer status, housing, employment, education are mentioned. (Was occupation considered?)
- Also, I think that talking about ‘variations’ might be unnecessarily euphemistic at this point. And i would suggest aiming to condense the key messages of the paper. So perhaps along the lines of:
  “Inconsistent recording of patient data means that it is not possible to assess variations in social prescribing patterns that may exist across patient groups. To identify and monitor inequalities in social prescribing, consistent recording of data is required on relevant patient factors (these may include disability, carer status, housing, employment, education, as well as the more reliably-recorded age, gender, ethnicity). Preliminary results suggest that inequalities exist in social prescribing, with some patient groups underrepresented in referrals.”

On Presentation of data

- Table 1 made little sense to me and it’s content might be better explained in a short paragraph.
- Table 2 is very busy and unclear. I think part of the problem is that the labels on the columns are inadequate – they do not stand alone to convey the information of the column’s content.
- Table 3 might not be necessary? It reveals only that differences exist between practices and regions, but these differences are not explained or explored further by the present study.

Data

- The inclusion of IMD data seemed unhelpful. From what I understand, this data was only obtained at the practice level (not for individual patients), which makes it an extremely blunt tool in the analysis. If I have misunderstood and the authors feel it worth including, then they should explain why, and explain the IMD score – specifically, the point that a lower IMD score (or IMD decile in this case) denotes greater deprivation.

Thanks for the opportunity to read and comment on this paper. As mentioned, i think it makes an important contribution.

Is the work clearly and accurately presented and does it cite the current literature?

Partly

If applicable, is the statistical analysis and its interpretation appropriate?

Yes

Are all the source data underlying the results available to ensure full reproducibility?

No

Is the study design appropriate and is the work technically sound?

Yes

Are the conclusions drawn adequately supported by the results?

Yes

Are sufficient details of methods and analysis provided to allow replication by others?

Yes

Reviewer Expertise:

I feel able to comment on the measuring and monitoring of healthcare inequalities, and on relevant data recording.

I confirm that I have read this submission and believe that I have an appropriate level of expertise to confirm that it is of an acceptable scientific standard, however I have significant reservations, as outlined above.

# Response

**Published:** 2023-08-24

**Authors:** Rachel Al-Izzi

**Affiliations:** University of Central Lancashire, UK

Thank you for taking the time to review our paper. We are grateful for the feedback you provided and will outline our response to your comments here.

 On your comments about the 'Abstract' section lacking clarity, including some ambiguous phrases, repetition in the results section, and the need to specifically name the demographics which were poorly recorded:

 Thank you for your comment. We reviewed the abstract and made various minor changes for clarity in the 'Background' section which now reads: 'Social prescribing (SP) is part of universal personalised care and available to everyone in the UK National Health Service. However, emerging evidence suggests access disparities in social prescribing. This study aimed to investigate the feasibility of using primary care records to access and analyse data on social prescribing. Our secondary aim was to compare characteristics of patients who are offered referral, referred to or decline referral for social prescribing to explore possible inequalities in access to social prescribing.'

 Also in the results section: 'GP referral data showed inconsistent recording of wider determinants of health. Patient age, sex and mental and physical health conditions were consistently recorded. Other variables (marital status, interpreter need, education, disability, sexual orientation, non-English language, nationality) were recorded only sporadically and could not be used for analysis.  Practices varied in referral patterns.'

 Conclusion section: 'Recording discrepancies meant that many patient factors could not be used to assess trends in social prescribing referrals and declines. Primary care data recording must be consistent to understand SP referral trends and inequalities relating to the wider determinants of health. Preliminary results suggest some patient groups may be underrepresented in SP referrals, however this requires further investigation.'

 Thank you for your input on this. We hope these edits have addressed your points on clarity and the importance of our findings on recording wider determinants of health.

 Data Presentation

 On your comment on Table 1 being unclear:

 Thank you for your comment. Table 1 has now been revised for clarity. All excluded fields are contained in one column titled 'other fields excluded due to missing data' and general and mental health columns have been combined. We hope this has made this table clearer.

 On your comment on Table 2 being too busy, unclear and the column labels being inadequate:

 Thank you for your insight. Table 2 has now been revised to align with your comments. I have removed the percentages columns and the 'referred' column and reduced to three columns headed: 'Referred/offered/declined (all codes)', 'Comparison practice population %' and 'Declined code only (% within demographic)'. Percentages and total numbers have been included in one column instead of listed separately as previous. We hope that in doing this we have reduced the busyness of the table and created column labels which are more descriptive of the information in the table.

 On your comment on Table 3 being unnecessary and not interpreted in the text:

 Thank you for your comment. We agree we did not fully explore this area of the study. We acknowledge your comment but opted to keep Table 3 to reflect the differing referral patterns. We have added to the 'Limitations' section to reflect the relevance of this. The section now reads: 'The heterogeneity in referral patterns and variations in recording may not have given us a full picture of declines however the data has highlighted possible differences in referral and decline rates for some population groups. Variation in coding behaviour means that this data cannot be used as a fully reliable indicator of referral patterns. Declines might not be consistently recorded.'

 On your comment on the IMD decile information included, ?what information we had access to and a description of the meaning of IMD decile:

 Thank you for your comment, we appreciate there was a lack of clarity in our paper on the IMD. We hope we have rectified this by adding to the 'IMD decile' paragraph in the 'Data analysis' section: 'Index of Multiple Deprivation deciles indicate the deprivation level for the postcode associated with each patient. Decile 1 is the 10% most deprived areas in England and decile 10 is the 10% least deprived areas in England. IMD decile is available for all 3086 patients in our dataset, but in the comparison data pulled from practice populations, IMD data was only available at the GP practice level.'

# Reviewer report: Reviewer response for version 1

**DOI:** 10.3310/nihropenres.14451.r29420

**Published:** 2023-06-23

**Authors:** Denise Ann Taylor

**Affiliations:** 1 Victoria University of Wellington, Wellington, Wellington, New Zealand

Thank you for asking me to review this paper on whether referrals to social prescribing can be used to detect bias/inequity on the types of people attending. I have the following comments to make on an overarching level to start.

1. In many places, people can self-refer to social activities, and/or they are introduced to social prescribing by friends or neighbours who are taking part in these activities. I remain unconvinced these would be entered into a GP database.
2. The authors report not finding diagnoses of mental illness. This is the point of social prescribing that social activities are attended by people with social determinants of low mood or mental health or anxiety (for example) before a frank mental illness is diagnosed. It is about preventing the worsening of mental health and encouraging engagement in activities that improve the individual's mood and mindset by engaging with others that seem the same as them. That is not to say I haven't seen people with severe mental health diagnoses taking part in social activities, as I have been involved in such a project. The activities take the individual outside of their distress by offering an alternative activity to focus on.
3. The focus of social prescribing is to de-medicalise the situation and not classify life events as illnesses because we know that antidepressants are of little benefit to any person with mild to moderate depression (NICE NG222), but they do add to the increased risk of suicide ideation, bleeding (especially if a person is taking aspirin for cardiovascular disease) and weight loss and nausea in the short-term, amongst other adverse effects.
4. Social prescribing offers a non-pharmacological approach to addressing low mood and also supports individuals in building links with like-minded people to feel included back into society and their local community.
5. One might expect older people to be more frequent attendees at social prescribing facilities as ageing is a well-known mood depressor in its own right, and many older people may be property-rich but cash poor, especially if their partner has passed away, leading to isolation and loneliness and other precursors of poor mental health.

I think this was an interesting study, but it seemed there was a lack of understanding of how social prescribing works at times. I note that the study was completed in a geographical place where I know little about health deficits, and findings may be completely different in other geographical areas. This is because social prescribing is generally built to suit the needs of local residents, young and old.

 In terms of publication, the authors should consider whether the indicators they chose are appropriate for capturing social prescribing, which is aimed at de-medicalisation. Furthermore, to actually look at deficits/inequity, when referring people to social prescribing, it may have been appropriate to select known areas of deprivation against geographical areas with less social deprivation. This may have been the case, but I don't know the geographical areas sufficiently well to judge this.

Is the work clearly and accurately presented and does it cite the current literature?

Partly

If applicable, is the statistical analysis and its interpretation appropriate?

I cannot comment. A qualified statistician is required.

Are all the source data underlying the results available to ensure full reproducibility?

No

Is the study design appropriate and is the work technically sound?

Partly

Are the conclusions drawn adequately supported by the results?

Partly

Are sufficient details of methods and analysis provided to allow replication by others?

Partly

Reviewer Expertise:

Social prescribing pathways for mental health and other activities that are necessary to address inequity in the local population that I have worked and researched in. Health and Diversity and inequity.

I confirm that I have read this submission and believe that I have an appropriate level of expertise to confirm that it is of an acceptable scientific standard, however I have significant reservations, as outlined above.

# Response

**Published:** 2023-08-24

**Authors:** Rachel Al-Izzi

**Affiliations:** University of Central Lancashire, UK

Thank you for taking the time to review our paper. We are grateful for the feedback you provided and will outline our response to your comments here.

 On your comment (1) about the many models of social prescribing:

 Thank you for this comment and we appreciate that GP databases do not record all social prescribing activity. We have edited the 'Introduction' section (3rd paragraph) to clarify this as follows: 'Although SP is often accessed through a GP referral, there is no one standard model. Other agencies can refer to social prescribing and although link workers are usually involved, some models exist without them.  It is therefore a complex non-medical intervention with numerous interacting components from systems and services to providers and patients. In this paper we will examine GP practice referrals to SP.'

 On your comments (2-5) on mental illness, general health conditions, the de-medicalised and non-pharmacological approach of social prescribing, and the fit of older people within the service:

 Thank for your comments 2-5 and your helpful insights into SP. We understand the role of SP is not focused on general or mental health diagnoses but feel our aim has been misunderstood. The relevance of health and mental health in this paper was to investigate which groups were accessing social prescribing or declining. These variables were used to explore if those with a diagnosis were being referred by GPs or were declining (potentially indicating inappropriate referrals or barriers to access). We have added a passage in the 'Data collection' section following Table 1 to clarify this: 'These Demographics were chosen to reflect key social determinants of health (individual personal circumstances that affect health and wellbeing). The aim was to understand representation of these factors in our dataset and how they might influence rates of decline.'

 On your comment (I think this was an interesting study, but it seemed there was a lack of understanding of how social prescribing works at times. I note that the study was completed in a geographical place where I know little about health deficits, and findings may be completely different in other geographical areas. This is because social prescribing is generally built to suit the needs of local residents, young and old.)

 Thank you for your comment: we hope this has been addressed in our response to your comment (1) above, that our focus was on a specific SP pathway (GP practice referrals).

 On your comment about our chosen variables and the importance of comparing areas of higher deprivation with areas of lower deprivation:

 Thank you for your comment. We chose our variables based on the social determinants of health and have added a line to reflect this in the 'Data collection' section following Table 1: 'These Demographics were chosen to reflect key social determinants of health (individual personal circumstances that affect health and wellbeing). The aim was to understand representation of these factors in our dataset and how they might influence rates of decline.' We considered factors which might influence take up/decline. We agree that it would have been really useful to explore both areas of deprivation and those which are less deprived. However, due to study time limitations we had to accept practices that had the capacity and willingness to participate in the study. This is definitely something we can consider in future studies.

# Reviewer report: Reviewer response for version 1

**DOI:** 10.3310/nihropenres.14451.r29088

**Published:** 2023-04-26

**Authors:** Andrea E. Williamson

**Affiliations:** 1 Undergraduate Medical School, College of Medicine, Veterinary and Life Sciences, University of Glasgow, Glasgow, Scotland, UK

This is an important topic in terms of understanding better the context of social prescribing in general practice and also the contribution it might make to tackling or exacerbating health inequalities. The sampling is pragmatic appropriate and justified. The data is not publicly available which is normal in the UK for data of this nature. Excellent General Practice research! Thank you!

 The main issue that needs addressed is that the paper currently conflates coding behaviour and action. The data are likely to reflect BOTH coding and referral behaviour and it is not possible with this data set to know what the gap is. Mainly, the patient is offered/is referred etc., but the clinician does not code it. This needs to be made clear at certain points in the paper - see suggestions for change below in the specific minor section.

 To conceivably try and address that gap, you could in future research match CLW data from the CLW organisations to the practice data – as this is likely to be much more accurate and would help interrogate this gap in more depth. I am not aware of any papers that have done that yet. It makes sense that organisations who are funded to provide this service will be more likely to have accurate data on referrals. Of course that sheds no light on ‘offered’ or ‘declined’ referrals.

**Minor comments:**

 Intro paragraph 3:

 There are now community links workers in services beyond general practice, including in mental health services. So although this statement about it mostly being in GP was true some years ago, the landscape has changed. Also SP is often, but not exclusively, delivered through CLW. For example, in many Inclusion Health settings, GPs, nurses pharmacists will do their own SP referrals as well as working with a CLW. In summary it is complex and many models and practices co-occur. Amend the intro to better reflect this please.

 Data Analysis Practice population - *recorded* as being exposed to SP rather than definitely being exposed, needs amended.

 Acceptance/declined rates - is this description of how the codes were used what you *assumed* GPs were doing with them? Feasibly some may code offer then also code accepted/declined in the same contact. Coding behaviours are variable by clinician and by any given day etc, as I’m sure you know. You need to state this is an assumption. The next paragraph goes onto describe some examples of this complexity. Suggest you frame it thus. Helps the reader understand the complexity of the coding context.

 Typo in table 1 mental ‘ealth’

 Also the headings do not make sense. Employment, housing are social determinants of health. Could include carer role there too. Suggest have a heading for that, then general health include mental illness. Have excluded all as one column - at the moment that reads strangely and I find myself thinking there is a typo and one might actually be included (even though that’s not the case).

 Say in the text what IMD 1 and 10 means as otherwise the reader cannot readily interpret table 2.

Is the work clearly and accurately presented and does it cite the current literature?

Yes

If applicable, is the statistical analysis and its interpretation appropriate?

Yes

Are all the source data underlying the results available to ensure full reproducibility?

No

Is the study design appropriate and is the work technically sound?

Yes

Are the conclusions drawn adequately supported by the results?

Partly

Are sufficient details of methods and analysis provided to allow replication by others?

Yes

Reviewer Expertise:

Academic GP, Inclusion Health and tackling health inequalities expertise, experience of using large GP data sets in research.

I confirm that I have read this submission and believe that I have an appropriate level of expertise to confirm that it is of an acceptable scientific standard, however I have significant reservations, as outlined above.

# Response

**Published:** 2023-04-27

**Authors:** Rachel Al-Izzi

**Affiliations:** University of Central Lancashire, UK

Thank you for your report and comments which were really useful feedback for us. We've taken them on board and will apply them once our paper is re-opened for amending. We hope you're happy with the revised version when it comes out!

# Response

**Published:** 2023-08-24

**Authors:** Rachel Al-Izzi

**Affiliations:** University of Central Lancashire, UK

Thank you for taking the time to review our paper. We are grateful for the feedback you provided and will outline our response to your comments here.

 On your first comment for coding behaviours vs actions and whether clinicians are recording accurately, and your query in the minor comments section on duplicate codes: 

 Thank you for your comment. There were no cases where multiple codes were given to one patient but we had not clarified this in the text so we are grateful for your input here. We have now rectified this by adding text to the ‘Acceptance/decline rates’ paragraph of the ‘Data analysis’ section: 'The dataset was checked for duplicate patients: none were found and only one code was associated with each patient record.' Re coding behaviours we added text reading: 'Coding patterns were variable and it seems use of these codes differs by clinician/practice. This is detailed in our ‘Limitations’ section at the end of this paper.' We have also added further detail to our ‘Limitations’ section reading: 'Variation in coding behaviour means that this data cannot be used as a fully reliable indicator of referral patterns. Declines might not be consistently recorded.'

 On your comment for matching CLW data with our referral data:

 Thank you for your comment. This is definitely a worthwhile area to explore in the future.

 On your comment regarding the SP landscape and the many models which exist:

 Thank you for your comment which we've taken on board. The ‘Introduction’ section has now been amended to read: 'Although SP is often accessed through a GP referral, there is no one standard model. Other agencies can refer to social prescribing and although link workers are usually involved, some models exist without them.  It is therefore a complex non-medical intervention with numerous interacting components from systems and services to providers and patients.' We hope this better reflects the current UK landscape of SP.

 On your comment (Data Analysis Practice population - recorded as being exposed to SP rather than definitely being exposed, needs amended):

 For clarity this has now been changed to: 'All three SP codes (‘referred’, ‘declined’ and ‘offered’ as outlined in the Data Collection section above) were used to present the total numbers of those who had been ‘offered/declined’ social prescribing through their GP.' This now also reflects the wording used throughout the text and in the tables.

 Thank you for drawing attention to the typo which has now been corrected.

 On your comment about Table 1 (unclear heading and unnecessary extra columns):

 Thank you for your insight - Table 1 has now been changed to reflect your comments with one 'excluded' column and one 'medical diagnoses' column.

 On your comment about IMD deciles and making this information clear to the reader:

 Thank you for your comment. In the IMD decile section under the 'Data Analysis' header, the following passage is now included to explain IMD deciles: 'Index of Multiple Deprivation deciles indicate the deprivation level for the postcode associated with each patient. Decile 1 is the 10% most deprived areas in England and decile 10 is the 10% least deprived areas in England.'
